# Serological biomarkers associate ultrasound characteristics of steatohepatitis in mice with liver cancer

**DOI:** 10.1186/s12986-018-0304-9

**Published:** 2018-10-05

**Authors:** Guozhen Cui, Robert C Martin, Xingkai Liu, Qianqian Zheng, Harshul Pandit, Ping Zhang, Wei Li, Yan Li

**Affiliations:** 1 Department of Hepatology, Cancer Center, The First Hospital of Jilin University, No. 71. Xinmin Street, Changchun, Jilin, 130021 China; 2 Division of Surgical Oncology, Department of Surgery, School of Medicine, University of Louisville School of Medicine, 511 S Floyd ST MDR Bldg, Louisville, KY 40202 USA; 3 Department of Hepatobiliary and Pancreatic Surgery, The First Hospital of Jilin University, Changchun, 130021 China; 4 Department of Pathophysiology, Basic Medicine College, China Medical University, Shenyang, 110122 China

**Keywords:** Ultrasound imaging, Serum biomarker, Steatohepatitis, Hepatocellular carcinoma

## Abstract

**Background:**

Non-alcoholic fatty liver disease (NAFLD) covers a spectrum of lesions ranging from steatosis to a complex pattern, nonalcoholic steatohepatitis (NASH). Ultrasonography provides important information on hepatic architecture for steatosis. NASH patients have an increased risk of hepatocellular carcinoma (HCC). Early detection of NASH is critical for clinicians to advise on necessary treatments to prevent the onset of HCC.

**Methods:**

We established a NASH-HCC mouse model using diethylnitrosamine as a carcinogen to induce HCC and a high-fat diet to induce metabolic disorders. Characteristics of ultrasound imaging and potential serum biomarkers were investigated for detection of steatohepatitis and HCC in mice.

**Results:**

The data suggested that ultrasound imaging of hyperechoic masses was potentially linked to the gross finding of HCC nodules, which was further confirmed by the histology. Positive correlation between serum fibroblast growth factor 15 and acoustic attenuation coefficient was found in mice with steatohepatitis. Combined with the serum markers, the increased acoustic attenuation coefficient could be a useful diagnostic parameter of ultrasound imaging for NASH detection.

**Conclusions:**

This study demonstrates that a combination of serum fibroblast growth factor 15 and acoustic attenuation coefficient could be a sensitive marker for steatohepatitis and to predict carcinogenic initiation and progression of HCC in mice. These results might help for the design of ultrasound and surrogate markers in screening NASH patients who could be at risk of HCC.

## Background

Non-alcoholic fatty liver disease (NAFLD), a metabolic disorder, covers a spectrum of hepatic lesions ranging from steatosis to a complex pattern of nonalcoholic steatohepatitis (NASH) [1]. The earliest stage of NAFLD is hepatic steatosis, which is characterized by the deposition of triglycerides (TGs) as lipid droplets in the cytoplasm of more than 5% of hepatocytes [2]. NASH is distinguished from simple steatosis by the presence of hepatocyte ballooning and hepatocyte cell death, inflammatory infiltration, and/or fibrosis [3]. Prevalence of NAFLD has been estimated around 25–30% in the general population [4]. NASH, the most extreme form of NAFLD, can progress to cirrhosis. About 10 to 29% of NASH patients develop cirrhosis within 10 years [5] and those patients with cirrhosis have an increased risk of hepatocellular carcinoma (HCC) [6]. Because of absence of noninvasive strategies for NASH diagnosis, liver biopsy becomes the gold standard technique to diagnose NASH and early liver fibrosis. Unfortunately, the prevalence of NASH continues to increase [7] but its incidence cannot be known accurately because of the impossibility of performing liver biopsy in the general population.

Over the past decade, there is a growing interest to find alternative noninvasive strategies, including computed tomography (CT), magnetic resonance imaging (MRI) and ultrasonography for NAFLD diagnosis [8]. Ultrasonography provides important information on hepatic architecture as the first imaging modality. Traditional ultrasound is an excellent diagnostic technique to reveal the presence of simple steatosis because hepatic fat deposition causes increased echogenicity on ultrasonography owing to intracellular accumulation of lipid vesicles. Fat deposition increases scatter including backscatter (signal), making the liver not only more echogenic but also more attenuating with decreased signal to noise ratio. In fact, ultrasonography is a relatively cheap and widely available and simplistic imaging technique for steatosis diagnosis. Although the sensitivity and specificity of grayscale ultrasound for the detection of simple steatosis are less compared to CT or MRI [9], ultrasound is often used as a technique for clinicians review images manually to make diagnostic decisions and identify the abnormalities present in the images.

Early detection of NASH is critical for clinicians to advise on necessary treatments to prevent the onset of HCC. Ultrasonography has 60–94% sensitivity and 84–95% specificity for detecting hepatic steatosis [10], however it is neither sensitive nor specific to reveal NASH and fibrosis, except in advanced stages where signs of cirrhosis are evident. Complementary approaches have been developed by assessment of serum biomarkers along with the liver architectures using ultrasound imaging [11]. These approaches may achieve more accurate information of liver pathology for clinicians without the need for liver biopsy and additional CT or MRI scans, replacing those relatively painful and more expensive approaches. However, the data to use ultrasound and serum biomarkers to study the characteristics of NASH is largely absent in both patients and experimental animals.

Recently, we established a mouse model with high-fat diet (HFD) feeding for a time course study. The results showed that fatty liver was induced within 2 months after HFD administration. Based on this HFD model, we further established a steatohepatitis-HCC mouse model using diethylnitrosamine (DEN) as carcinogen for steatohepatitis and HCC development. The results demonstrated stepwise changes from simple steatosis to steatohepatitis to HCC. In this current study, we propose to use our NASH-HCC mouse model to investigate characteristics of ultrasound imaging for steatohepatitis and liver cancer in mice within a time course development. The aim of this study was to provide insight into the ultrasound imaging features associated with potential serum biomarkers during the progression from fatty liver to NASH and HCC. Understanding the characteristics of ultrasound imaging and associated serum biomarkers during steatohepatitis-HCC carcinogenetic transformation could provide useful information and develop a valuable tool in the future to screen NASH patients who are at HCC risk.

## Methods

### Animals and NASH-HCC procedure

C57L/J mice, obtained from Jackson Laboratory (Bar Harbor, ME), were housed four per cage, given commercial chow and tap water, and maintained at 22 °C on a 12-h light/dark cycle. Sexually matured male and female mice were set as breeding pairs for offspring. At 15 days of age, male mice, yielding the F1 generation, were administered 75 mg/kg body weight DEN (Sigma-Aldrich, St. Louis, MO) by intraperitoneal injection (i.p.). When aged at 1 month, the mice were fed with HFD (Rodent Diet with 60% kcal% fat, D12492, Research Diets, Inc., New Brunswick, NJ) or Control Diet (CD) with 10% kcal% fat (D12450B, Research Diets, Inc., New Brunswick, NJ). The animals without DEN treatment were fed with HFD or CD as controls. The animals underwent ultrasound imaging monthly. The animals were sacrificed at 2 months, 6 months and 10 months. At respective time points for sacrifice, gross anatomy of the liver was examined and HCC nodules were counted. Serum and hepatic tissues were harvested from all animals for further analysis. The animal procedures were approved by the Institutional Animal Care and Use Committee of University of Louisville, which is certified by the American Association for Accreditation of Laboratory Animal Care.

### Histopathology and NASH scoring

Gross liver anatomy observation was performed and the HCC nodules and masses were counted for each mouse. The liver lobes were sliced and the number of macroscopic foci nodules ≥1 mm was recorded using a magnifier for each animal. The harvested liver tissues were fixed in 10% buffered formalin for 24 h and transferred to ethanol for dehydration, and then the  tissues were embedded in paraffin. Serial sections of 5-μm were mounted onto glass slides for histopathological analysis. Hematoxylin and eosin (H&E)-stained slides were obtained for each animal. Evidence for NASH was identified in hepatic parenchyma in terms of histological changes, including steatosis, infiltration of inflammatory cells in acinar zone, and form of ballooning. HCC was diagnosed by the main hallmark of HCC, i.e., its resemblance to the normal liver in both its plate-like growth and its cytology. HCC cells, however, show different degrees of hepatocellular differentiation, ranging from well to poorly differentiated, which are based upon the architectural and cytologic features. Cytologically, tumoral hepatocytes are polygonal, displaying an eosinophilic granular cytoplasm, rounded nuclei and prominent nucleoli. The Histological Scoring System for NASH is reported by the Pathology Subcommittee of the NASH Clinical Research Network [12]. This scoring system is calculated by the sum of scores of steatosis (0–3), lobular inflammation (0–3) and hepatocyte ballooning (0–2). The scoring is conducted as follows: Steatosis: 0, < 5%; 1, 5–33%; 2, > 33%; 3, > 66. Lobular Inflammation: 0, no foci; 1, < 2 foci/200X; 2, 2–4 foci/200X; 3, > 4 foci/200X. Hepatocyte Ballooning: 0, no balloon cells; 1, 1–5 balloon cells/200X; 2, > 5 balloon cells/200X.

### Biochemical analysis

To analyze the liver injury and metabolic abnormalities in the liver, serum plasma alanine aminotransferase (ALT), serum glucose, serum insulin, serum and liver triglyceride (TG) were determined. The serum ALT was measured using an ALT infinity enzymatic assay kit (Thermo Fisher Scientific Inc., Waltham, MA), according to the instruction provided. Serum insulin was detected using an Ultra Sensitive Mouse Insulin ELISA Kit kit (Crystal Chem USA, IL), according to the instruction provided. Serum glucose assay was performed using a Sigma assay kit (Sigma-Aldrich Company, MI). TG assay was performed with TG assay kit (Cayman Chemical Company, CA).

### Fibroblast growth factor15 (FGF15) assays

The collected sera were centrifuged for 15 min at 1000 x g within 30 min. The serum samples were standardized corresponding to the total protein concentration. FGF15 protein levels were detected by an ELISA assay using a DuoSet ELISA Ancillary Reagent kit 2 (R&D Systems, Inc. Minneapolis, MN). In brief, 100 μL dilution of FGF15 protein standards (0–2 ng/ml) and serum samples with capture antibody was coated in the 96-well microplate overnight at 4 °C After washing, the coated wells were adding 200 μL blocking buffer (1% BSA) for at least 2 h at room temperature. One hundred microliter anti-FGF15 goat IgG (AF6755) in Reagent Diluent was applied overnight at 4 °C, and then 100 μL donkey anti-goat IgG-HRP in Reagent Diluent was applied 2 h at room temperature. Substrate Solution was added for 30 min at room temperature, and stopped by Stop Solution. The OD value of each well was read using a plate reader at 450 nm. Concentration of the FGF15 was analyzed based on standard curve.

### Ultrasound imaging and acoustic attenuation

All animals were food deprived for 12 h before ultrasound. After induction and maintenance of anesthesia with 5% and 2% isoflurane, mice were shaved on abdomen, faced up and abdominal ultrasound examination was performed. Ultrasound images were acquired by professional medical experts using a high-frequency ultrasound imaging (VisualSonics 2100, Toronto, Ontario, Canada) with bandwidth of MS250 and the fundamental frequency of 30 MHz using Vivo Lab software (version 3.0.0) for analysis. The contrast ratio and light intensity were set up as 60% and 50%, respectively. Based on the feature of the acoustic attenuation in the tissues, various dissemination patterns for ultrasound could be shown in the livers of steatohepatitic mice or simple steatotic mice. On the transverse section of ultrasound images, two small circles (about 1mm^2^) were selected to avoid the blood vessel, bile duct and HCC nodules, if any. The length between the two circles was about 5 mm. Because acoustic attenuation showed as exponential function, acoustic attenuation coefficient “a” was calculated using the following equation, *a* = (p*A*1 – p*A*2) / (*L* × *F*), in which p*A*1 represented the averaged pixel value of circle 1, which was near the source of ultrasound, while p*A*2 represented the averaged pixel values of circle 2, which was far away from the source of ultrasound; L represented the length between the two circles; and F represented the frequency of the probe. Using this equation, the acoustic attenuation coefficient “a” was calculated in all the mice diagnosed with simple steatosis and steatohepatitis as well as controls. The dynamic range was fixed to 10 dB and the Time Gain Compensation was kept constant throughout the procedure.

### Statistical analysis

Data were collected from repeated experiments and were presented as mean ± SD. ANOVA was used to determine if difference exists. If so, a post-hoc Tukey’s test was used for analysis for the difference between groups. Spearman rank correlation coefficient (R) was used to analyze the correlation between acoustic attenuation coefficient (a) and serum markers using SigmaPlot analysis and graphing software. *P* values less than 0.05 were considered statistically significant.

## Results

### Fatty liver and HCC detection by ultrasonography

**Fig. 1:**
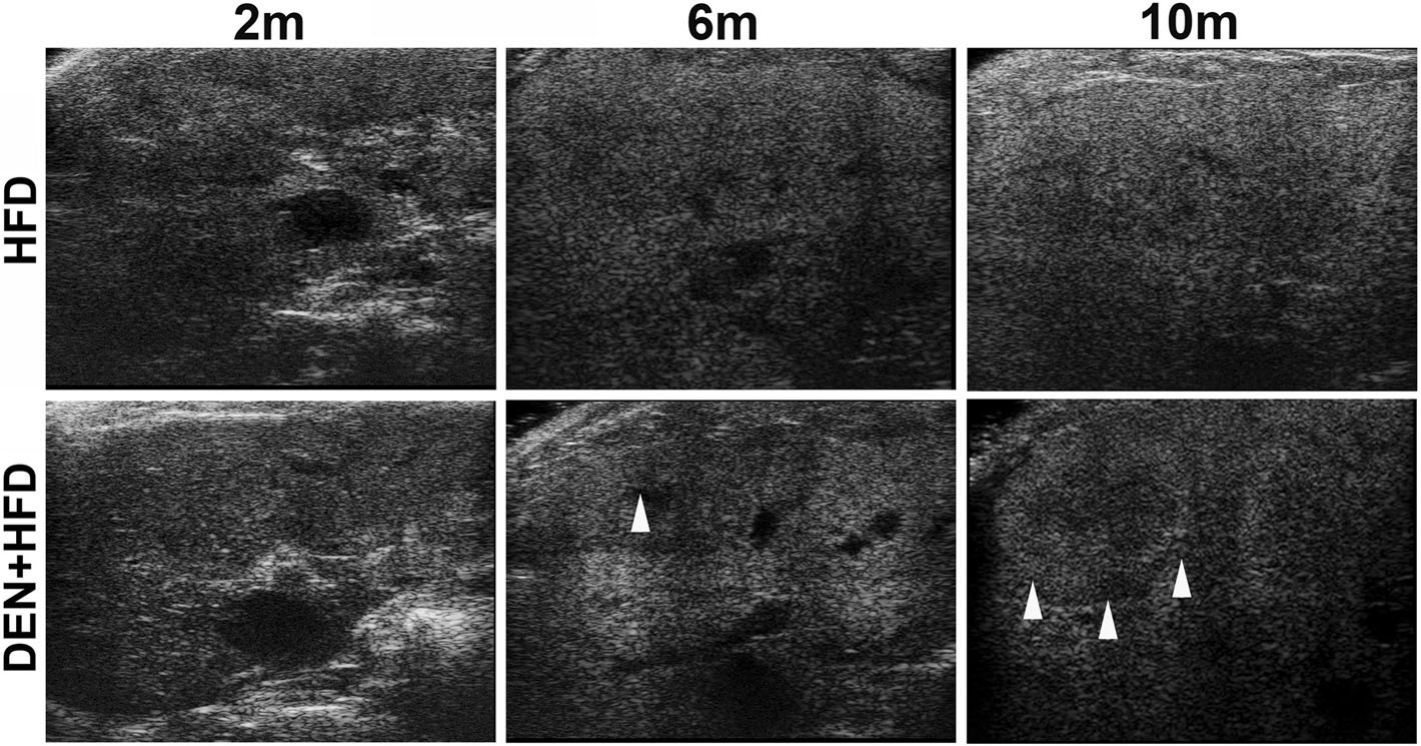
Representative ultrasound images from mice treated with HFD or DEN+HFD at 2 months, 6 months and 10 months. On visual pattern, increased intensity of the echogenicity due to fatty infiltration was found in the HFD treated mice and the DEN+HFD treated mice at month 2, month 6 and month 10. Hyperechoic mass detected in liver parenchyma in DEN+HFD treated mice at month 6 and month 10. m: month; white arrow head: hyperechoic mass

The ultrasound imaging was performed monthly on all mice to monitor alterations of ultrasonography during the developments of NASH and NASH-HCC. For the ultrasound features of liver on visual analysis, the liver parenchyma in the CD control mice showed a homogeneous echotexture with echogenicity, while significantly increased intensity of the echogenicity due to fat accumulation was detected in the HFD and DEN+HFD treated mice at month 6 and month 10. The portal vein diameter did not differ significantly between the HFD treatment group and DEN+HFD treatment group. In mice treated with DEN+HFD, hyperechoic masses in the liver parenchyma were found in 3 out of 6 at month 6, and in all 6 mice at month 10. In mice treated with DEN+CD, hyperechoic masses in the liver parenchyma were not found at month 6, but were found in 5 out of 6 mice at month 10. There were no hyperechoic mass detected in liver parenchyma in all the mice at month 2. The representative ultrasound images are shown in Fig. 1 from the treatment groups (HFD treatment and DEN+HFD treatment) at month 2, month 6 and month 10. The hyperechoic mass was consistent with the gross anatomy finding of the nodules on/in the sliced liver lobes, and the nodules were further confirmed as HCC tumors by histologic examination in H&E stained slides. The incidence of HCC is shown in Table 1.

**Table 1:** Incidence of NASH and NASH-HCC in mice

Time points	Group	n	NASH Incidence	HCCs Incidence
Month 2	CD	6	0(6)	0(6)
	HFD	6	0(6)	0(6)
	CD + DEN	6	1(6)	0(6)
	HFD + DEN	6	2(6)	0(6)
Month 6	HFD	6	0(6)	0(6)
	CD + DEN	6	2(6)	0(6)
	HFD + DEN	6	6(6)	3(6)
Month 10	HFD	6	0(6)	0(6)
	CD + DEN	6	3(6)	5(6)
	HFD + DEN	6	6(6)	6(6)

### Histological NASH scoring evaluation

As previously reported in patients with NAFLD, the score of ≥5 strongly correlated with a diagnosis of “definite NASH” whereas the score ≤ 3 correlated with a diagnosis of “not NASH” [12]. Using this human NAFLD Active Score system, histological scoring for all mice was performed on liver tissue slides with H&E staining. We modified the diagnosis of steatohepatitis in mice. A score of ≥5 defined as steatohepatitis, whereas a score of < 5 defined as non-steatohepatitis. The results showed that there was no steatohepatitis diagnosed in mice treated with CD or HFD. Although no typical steatosis was detected in most mice with CD + DEN treatment, developments of hepatitis and hepatic fibrosis rendered high histological scores of ≥5 in 1 mouse at month 2, 2 mice at month 6 and 3 mice at month10, respectively. In mice treated with DEN+HFD, obvious steatosis was found in all the mice at month 6 and month 10. Using the NAFLD Active Score system, steatohepatitis was diagnosed in 2 mice at month 2, and all 6 mice at month 6 and month 10, respectively. The mice with steatohepatitis diagnosed by NAFLD Active Score system are shown in Table 1.

### Gross anatomy, histology and ultrasound characteristics

**Fig. 2:**
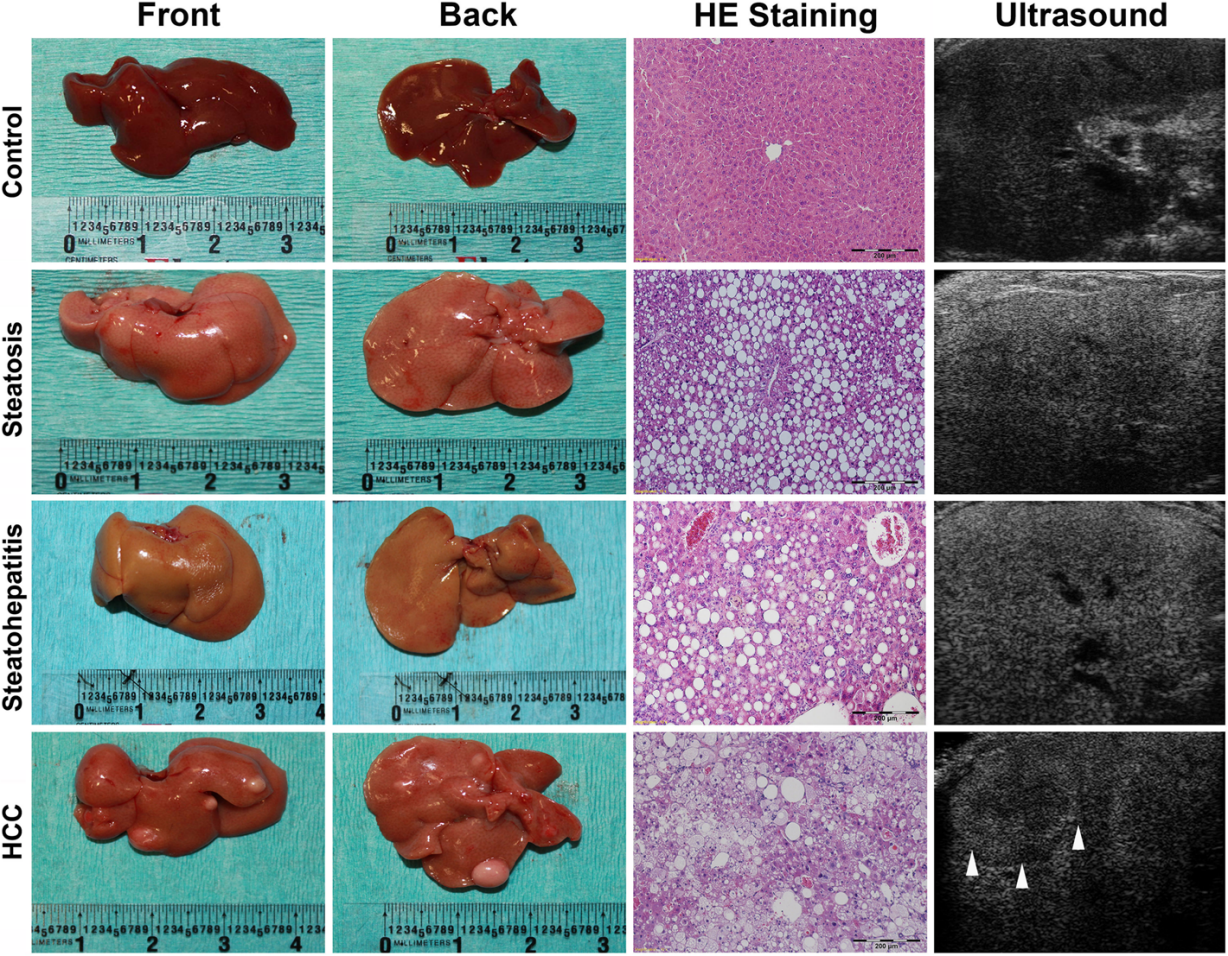
Representative gross anatomy, histology and ultrasound images from mice diagnosed with steatosis, steatohepatitis and steatohepatitis with HCC. The gross anatomy showed livers with a light-yellow color in the mice with simple steatosis, steatohepatitis and steatohepatitis with HCC. The HCC nodules were found in the mice treated with HFD + DEN. Histological changes indicated by H&E staining confirmed the simple steatosis as widely distributed lipid drops in hepatic parenchyma, steatohepatitis as inflammatory infiltration and hepatocyte ballooning, and HCC as destruction of normal hepatic architecture, plate-like growth and abnormal cytological structure of hepatocytes. The ultrasound images show increased intensity of echogenicity due to fatty infiltration in simple steatosis and steatohepatitis. Hyperechoic masses were detected in the ultrasound images from steatohepatitic mice with HCC. Scale bar size in HE staining = 200 μM

The representative gross anatomy of livers from the treatment mice as well as controls was shown in Fig. 2. With HFD treatment, fatty degeneration of the liver was observed, and the liver is smooth, of a light-yellow color, soft and easily torn. With DEN-HFD treatments, the features of fatty degeneration were also observed, while HCC nodules were also found throughout the liver in mice from month 10 (Fig. 2, right). The liver tissues from all treatment groups as well as controls were further analyzed by histological examination in tissue sections using H&E staining. The H&E staining confirmed the pathological changes for steatosis, steatohepatitis and HCC (Fig. 2, middle). Accordingly, a difference was shown in terms of intensity of the echogenicity on visual features from the liver with respect to the pathological changes for simple steatosis, steatohepatitis and HCC (Fig. 2, left). Because the variation of echogenicity was found from steatohepatitis, steatosis and control, the acoustic attenuation coefficient was further analyzed in the ultrasound images.

### Acoustic attenuation coefficient

**Fig. 3:**
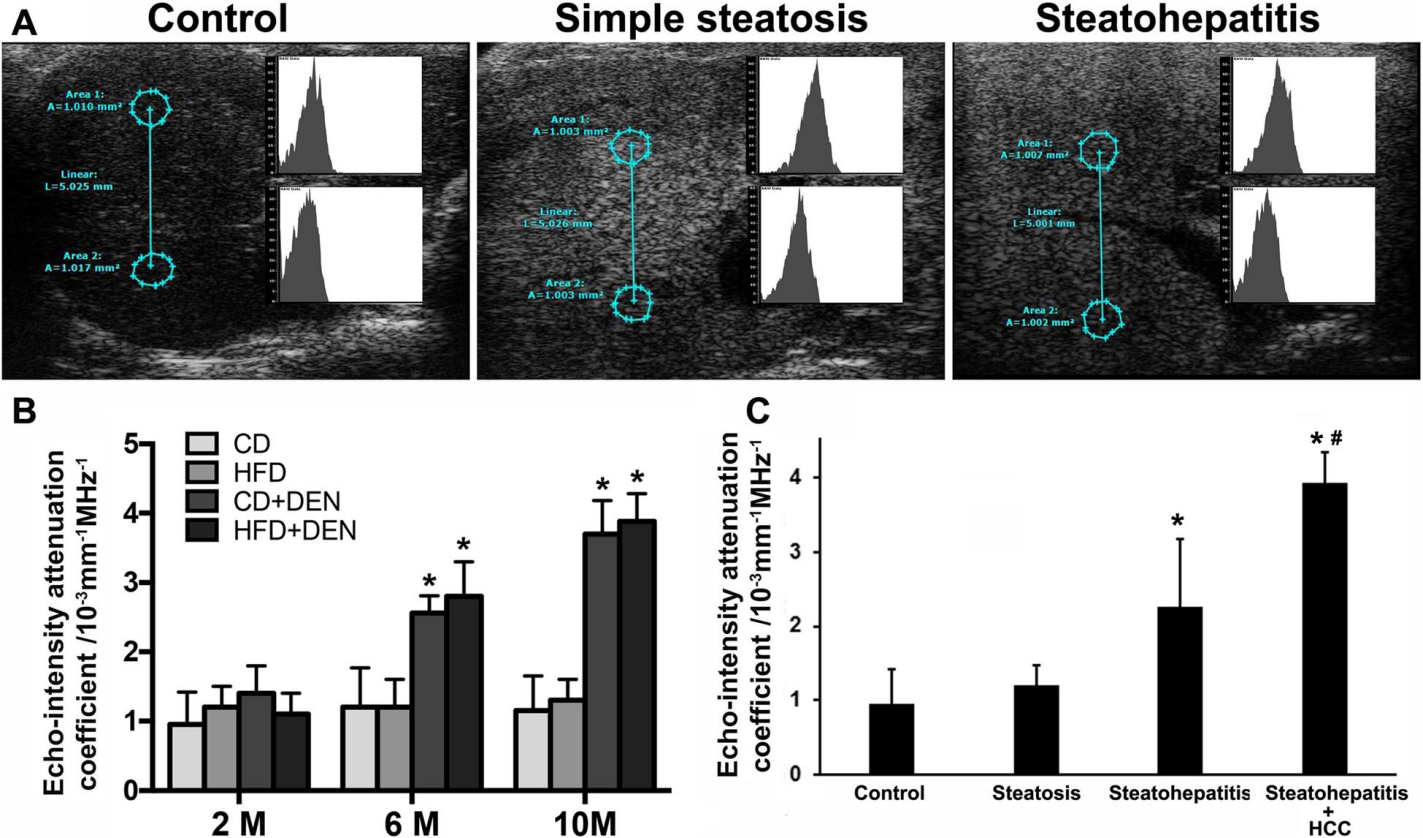
**a:** Schematic diagram for the liver acoustic attenuation coefficient “a” calculated from control, simple steatosis, and steatohepatitis. Area 1: near the source of ultrasound; Area 2: far away from the source of ultrasound; L: length between the two circles, Area 1 and Area 2. **b:** Acoustic attenuation coefficient in all mice from 4 groups at month 2, month 6 and month 10. **c:** Acoustic attenuation coefficient in mice diagnosed with steatosis, steatohepatitis and steatohepatitis with HCC. The data represent means ± SD. *, *p* < 0.05 vs CD control; #, *p* < 0.05 vs steatosis

The acoustic attenuation coefficient was calculated in all the mice as described in Methods. The Fig. 3a shows a schematic diagram for measurements of p*A*1, p*A*2 and length between the two circles in the representative ultrasound images from the mice of the control, simple steatosis and steatohepatitis groups. The results indicate that the acoustic attenuation coefficient was significantly increased in the mice with CD + DEN and HFD + DEN treatments at month 6 and month 10 (Fig. 3b). We further analyzed the acoustic attenuation coefficient from mice diagnosed as: simple steatosis, (NAFLD Active Score (NAS) < 4; steatohepatitis, NAS ≥ 5; and steatohepatitis with HCC. The results indicated that the acoustic attenuation coefficient significantly increased from the mice diagnosed with steatohepatitis and steatohepatitis with HCC compared to that from the normal control mice and simple steatotic mice. There was also a significant increase acoustic attenuation coefficient from steatohepatitic mice with HCC compared to that from steatohepatitic mice (Fig. 3c).

### Serological biomarkers and NASH score in mice

**Fig. 4:**
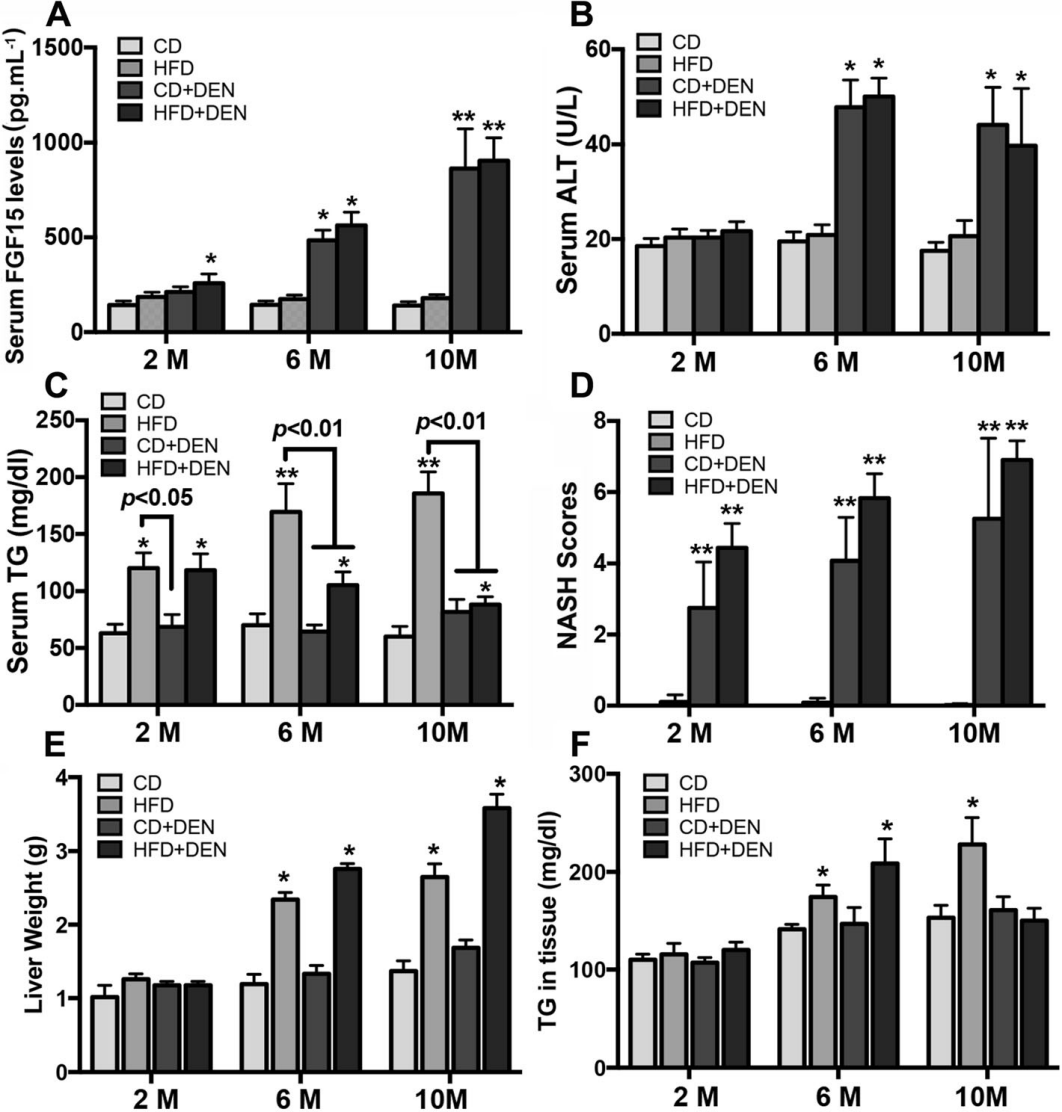
**a-c:** Analysis of serum levels of FGF15, ALT and TG in all mice from 4 groups at month 2, month 6 and month 10. **d-f:** Measurements of NASH score, liver weights and TG levels in tissues in all mice from 4 groups at month 2, month 6 and month 10. M: month. The data represent means ± SD. *, *p* < 0.05 vs CD control; **, *p* < 0.01 vs CD control

In the CD + DEN and HFD + DEN mice, the serum levels of FGF15 were significantly increased during disease development from month 2 to month 10. The serum levels of ALT were also significantly increased at month 6 and month 10. For the serum levels of TG, there was an increase in the mice treated with HFD and HFD + DEN compared to the CD control mice. The serum levels of TG were significantly increased in the HFD mice from month 2 to month 10; however, the increases of TG were blunt in the HFD + DEN mice at month 6 and month 10. The NASH score was further analyzed in all 4-group mice. The results indicated that the NASH scores were significantly increased in the CD + DEN and HFD + DEN groups at month 2, month 6 and month 10 (Fig. 4).

### Correlation analysis

**Fig. 5:**
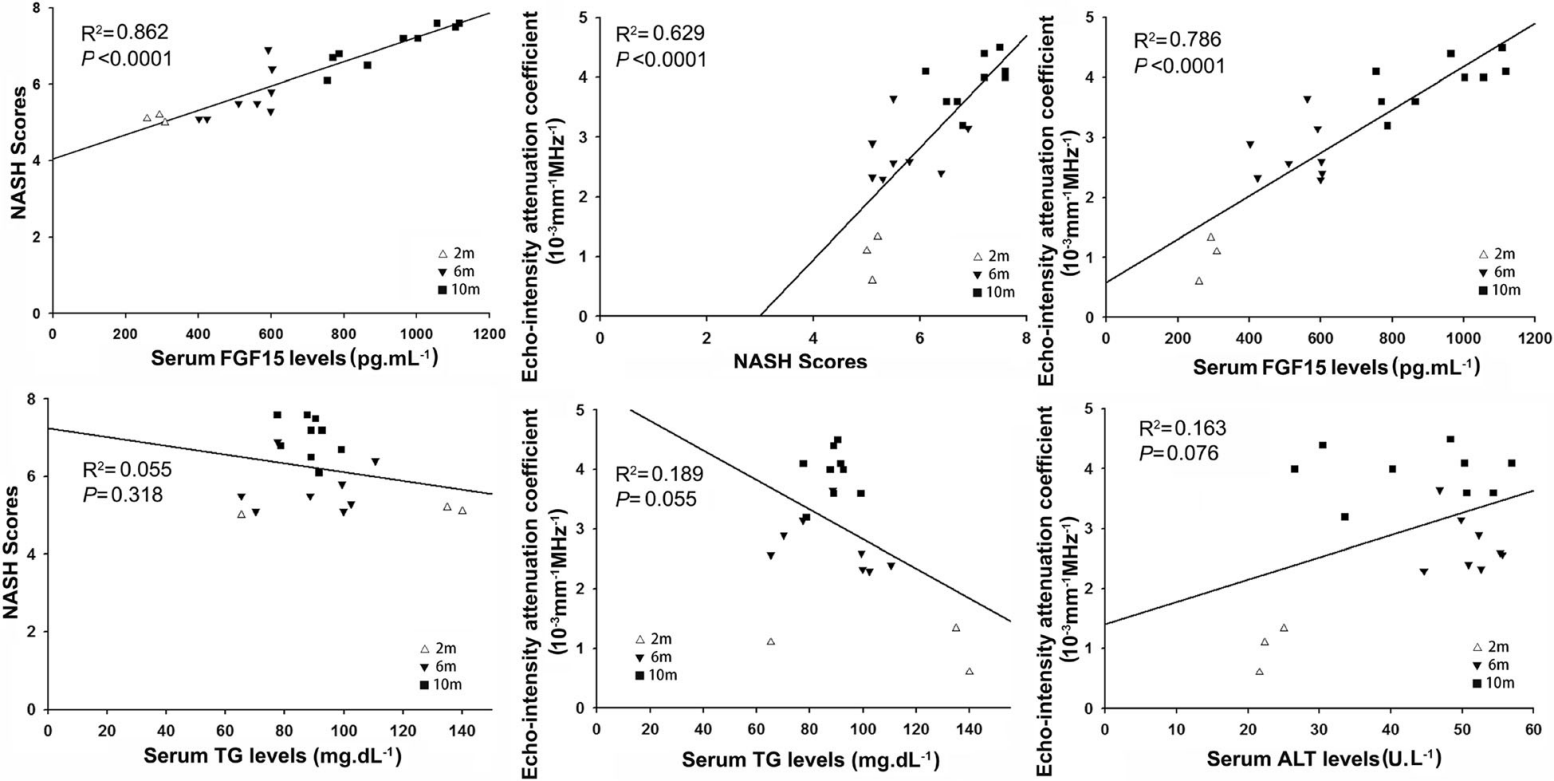
Linear regression analysis in paired data of the acoustic attenuation coefficient, NASH score and serological biomarkers from 20 mice with definite steatohepatitis. A significant positive correlation was found between NASH scores and serum FGF15; between acoustic attenuation coefficient and NASH scores; and between acoustic attenuation coefficient and serum FGF15. No correlation was found between NASH scores and serum TG; between acoustic attenuation coefficient and serum TG; and between acoustic attenuation coefficient and the serum ALT

The threshold values of the NAS, specifically NAS ≥ 5, was accepted as a surrogate for the histologic diagnosis of NASH [13]. Therefore, definite steatohepatitis was diagnosed in the mice with NAS ≥ 5 in current study. No mice with NAS ≥ 5 were found in the CD and HFD treated groups. In CD + DEN group, one mouse from month 2, two mice from month 6 and three mice from month 10 were with NAS ≥ 5 and diagnosed as definite steatohepatitis. In HFD + DEN group, two mice from month 2 and twelve mice from month 6 and month 10 were with NAS ≥ 5 and diagnosed as definite steatohepatitis. The paired data including NAS, acoustic attenuation coefficient, and the serological biomarkers were selected to perform linear regression and correlation analysis in all twenty mice diagnosed as definite steatohepatitis. As shown in Fig. 5, a statistically significant positive correlation was detected between NASH score and serum FGF15 (R^2^ = 0.862, *p* < 0.0001); between acoustic attenuation coefficient and NASH scores (R^2^ = 0.629, p < 0.0001); and between acoustic attenuation coefficient and serum FGF15 (R^2^ = 0.786, *p* < 0.001) (Fig. 5, upper). However, there was no correlation found between NASH scores and serum TG (R^2^ = 0.055, *p* = 0.318); between acoustic attenuation coefficient and serum TG (R^2^ = 0.189, *p* = 0.055); or between acoustic attenuation coefficient and serum ALT (R^2^ = 0.163, *p* = 0.076) (Fig. 5, lower).

## Discussion

In this study, we established animal models presenting simple steatosis, steatohepatitis and HCC, which were closely relevant to clinical patients. Using the models, the ultrasound features and serological biomarkers were analyzed to investigate the potential methodology for the diagnosis of steatohepatitis related to HCC in mice. Our data suggested that the ultrasound imaging of hyperechoic masses was linked to the gross finding of tumor nodule, which was further confirmed as HCC by histology. Although increased echogenicity was not considered to be specific for the histological changes of steatohepatitis, we found positive correlation between serum FGF15 and acoustic attenuation coefficient in the steatohepatitic mice, with increasing potential ultrasound values for NASH patients. This study demonstrates the ability to use ultrasound in combination with a serological biomarker to diagnose steatohepatitis in mice during steatohepatitis progression and carcinogenetic transformation.

As we know, definitive diagnosis of NASH is based on biopsy with the finding of steatohepatitis and exclusion of alcohol intake history (20 g or more of ethanol per day) and other liver diseases [14]. Although ultrasonography is the most widely used technique for fatty liver in clinical practice, it shows low specificity and low sensitivity if the liver is composed of less than 30% fat [15]. For other noninvasive strategies, CT is not superior to ultrasonography in the assessment of hepatic steatosis even though it provides quantitative and objective values in comparison with ultrasonography [16]. MRI-proton density fat fraction (PDFF) has been used to estimate the level of hepatic triglycerides, and is known as a perfect reference standard in terms of sensitivity and specificity for intrahepatic fat amount [17]. MRI-PDFF is even considered as a substitute for liver biopsy [18]. Unfortunately, it is difficult for both physicians and patients to choose high-cost equipment as a priority selection for using CT and MRI-PDFF. In addition, neither CT nor MRI-PDFF can discriminate between simple steatosis and steatohepatitis. In NAFLD population, detection of NASH is usually delayed since there is neither specific ultrasound imaging criteria nor serum surrogate markers for NASH diagnosis. In the current study, we found that increased intensity of echogenicity was linked to fatty liver but not specific for the histological changes of steatohepatitic mice. Interestingly, the acoustic attenuation coefficient increased much more in the steatohepatitic mice than that in the simply steatotic mice. Acoustic attenuation coefficient positively correlated with the increased serum marker of FGF15 in the steatohepatitic mice, but not the simply steatotic mice.

In fact, serum markers were previously developed, including Fatty Liver Index [19] and SteatoTest [20], which were used to detect simple steatosis. These serum markers are simple to obtain and may help physicians, but are not in widespread use because of the availability of the imaging tool-ultrasonography. Attempts to apply serological surrogate markers to distinguish steatohepatitis from simple steatosis were made in many studies in past decade. The recommended surrogate markers—including TNF-α, IL-6, ALT, Pantraxin3, Ferritin, and serum prolidase enzyme activity—predict hepatic inflammation [21]; however, most of these markers were not extensively and externally validated. Cytokeratin-18 was well studied as a single serological test for the diagnosis of NASH with the potential for screening for NASH; however, its sensitivity and specificity to predict steatohepatitis was not satisfactory [22]. In our current study, serological tests including FGF15, ALT, and TG were performed. The biomarkers of ALT and TG were well studied previously; however, the serological test for FGF15, the murine ortholog of FGF19, was first tested by our group to predict steatohepatitis. We selected this FGF15 marker because human FGF19 was found previously by our group as a potential marker to evaluate the risk of HCC in patients with NASH backgrounds [23]. In the current study, we found that the increased serum FGF15 positively correlated to the increased acoustic attenuation coefficient in mice during steatohepatitis progression and HCC development in mice treated with DEN and HFD. As previously reported, the well characterized function of FGF15/FGF19 was inhibition of hepatic bile acid synthesis by repressing cholesterol 7α-hydroxylase (CYP7A1) expression [24]. However, the serum level of FGF19 in NASH patients was controversial, in which FGF19 was reported as having no significant changes [25] or significant decreases [26] in serum in NASH patients. Our data showed increased serum FGF15 in the DEN+HFD mice during steatohepatitis and HCC development. Unlike the previous report, we found significantly increased serum FGF15 in the steatohepatitic mice that developed HCC in a later stage. We speculate that the up-regulated FGF15 could be in response to the underlying inflammatory alteration and carcinogenetic transformation in mice treated with the carcinogen-DEN, which induced hepatocyte proliferation and mitosis. Therefore, up-regulated serum FGF15 also could be a useful biomarker to detect HCC initiation, but further study is needed. Because a subpopulation of NASH patients might progress to liver failure, cirrhosis, and HCC [6], finding such a noninvasive predictor is critical for NASH patients who are at higher risk. The importance of our current study is the potential distinction between steatohepatitis and simple steatosis by ultrasound and serum markers. The increases of FGF15 and acoustic attenuation coefficient were specific to predict in the steatohepatitis-HCC mice. Nevertheless, the current study is limited by a lack of a model of steatohepatitis without using DEN as a control to investigate the serum FGF15 level in mice. Because HDF itself was unable to induce NASH in mice, future study is needed to establish a steatohepatitis model using a methionine-choline deficient diet.

## Conclusion

In conclusion, our study demonstrates that the combination of serum FGF15 and the acoustic attenuation coefficient could be a sensitive marker for steatohepatitis, and a possible predictor of carcinogenic initiation and progression of HCC in mice. These results might help for the design of ultrasound and surrogate markers in screening NASH patients who are at risk of HCC.
